# Natural Language Processing and Schizophrenia: A Scoping Review of Uses and Challenges

**DOI:** 10.3390/jpm14070744

**Published:** 2024-07-12

**Authors:** Antoine Deneault, Alexandre Dumais, Marie Désilets, Alexandre Hudon

**Affiliations:** 1 Department of Psychiatry and Addictology, Faculty of Medicine, Université de Montréal, Montreal, QC H3T 1J4, Canada; antoine.deneault@umontreal.ca; 2 Department of Psychiatry, Institut Universitaire en santé Mentale de Montréal, Montreal, QC H1N 3M5, Canada; alexandre.dumais@umontreal.ca (A.D.); mdesilets.iusmm@ssss.gouv.qc.ca (M.D.)

**Keywords:** schizophrenia, artificial intelligence, language, natural language processing, machine learning, schizoaffective disorder, psychosis

## Abstract

(1) Background: Approximately 1% of the global population is affected by schizophrenia, a disorder marked by cognitive deficits, delusions, hallucinations, and language issues. It is associated with genetic, neurological, and environmental factors, and linked to dopaminergic hyperactivity and neurotransmitter imbalances. Recent research reveals that patients exhibit significant language impairments, such as reduced verbal output and fluency. Advances in machine learning and natural language processing show potential for early diagnosis and personalized treatments, but additional research is required for the practical application and interpretation of such technology. The objective of this study is to explore the applications of natural language processing in patients diagnosed with schizophrenia. (2) Methods: A scoping review was conducted across multiple electronic databases, including Medline, PubMed, Embase, and PsycInfo. The search strategy utilized a combination of text words and subject headings, focusing on schizophrenia and natural language processing. Systematically extracted information included authors, population, primary uses of the natural language processing algorithms, main outcomes, and limitations. The quality of the identified studies was assessed. (3) Results: A total of 516 eligible articles were identified, from which 478 studies were excluded based on the first analysis of titles and abstracts. Of the remaining 38 studies, 18 were selected as part of this scoping review. The following six main uses of natural language processing were identified: diagnostic and predictive modeling, followed by specific linguistic phenomena, speech and communication analysis, social media and online content analysis, clinical and cognitive assessment, and linguistic feature analysis. (4) Conclusions: This review highlights the main uses of natural language processing in the field of schizophrenia and the need for more studies to validate the effectiveness of natural language processing in diagnosing and treating schizophrenia.

## 1. Introduction

Schizophrenia, a psychiatric disorder affecting approximately 1% of the global population, is marked by cognitive impairments, hallucinations, delusions, thought disorders, behavioral changes, and negative symptoms persisting most of the time for at least six months [1,2,3]. Lack of drive, a lowered capacity for pleasure, and a limited ability to express emotions are examples of the negative symptoms of schizophrenia that can seriously impede day-to-day functioning and quality of life [2,3]. These symptoms frequently show up as social disengagement, apathy, and a reduction in speech, which makes it difficult for the affected person to participate in daily activities or uphold relationships [2,3]. Current research into hallucinations and delusions suggests that these altered perceptions of reality are linked to dopaminergic hyperactivation in the mesolimbic system [4,5,6]. Schizophrenia frequently causes cognitive impairment that includes severe deficiencies in several areas, including language [7,8]. Various genetic, neurological, and environmental variables are understood to induce cognitive impairment in individuals with schizophrenia [9]. A person’s genetic predisposition is important because different genes influence how the brain develops and functions [10]. Neural pathways essential for cognitive functions are disturbed by neurotransmitter dysregulation, especially when it comes to dopamine and glutamate [11]. Cognitive deficiencies are also influenced by functional brain abnormalities such as hypo-frontality and poor connection, as well as structural brain abnormalities such as lower gray matter volume in important locations such as the hippocampus and prefrontal cortex, as observed in schizophrenia [12,13]. Neurodevelopmental variables are also very important; these include problems in pregnancy and early childhood that are caused by infections in the mother, starvation, and stress [14]. Additionally, environmental stresses like early-life trauma, social adversity, and substance addiction increase impairments in cognition caused by inflammation and oxidative stress, which can harm brain cells and are thought to be risk factors of psychotic disorders such as schizophrenia [15,16].

Recent studies have highlighted significant language deficits in patients diagnosed with schizophrenia, providing a deeper understanding of the nuances of these impairments [17]. Generally, individuals with schizophrenia demonstrate lower verbal productivity, evident in their production of fewer total words and reduced lexical diversity [18]. This is often accompanied by lower verbal fluency, characterized by frequent pauses, hesitations, and difficulties in maintaining a smooth flow of speech [18,19]. These disruptions in fluency can manifest as prolonged silences or repetitive non-communicative sounds, which contribute to the overall communication difficulties faced by these patients [20]. For example, these individuals frequently exhibit disjointed and fragmented speech patterns, where their discourse lacks logical organization and coherence [18,19,20]. This can result in abrupt topic shifts, inconsistent use of words, and difficulty in maintaining a clear narrative structure. Such semantic incoherence not only impairs effective communication but also reflects the underlying cognitive disorganization associated with schizophrenia [20]. Recent cross-linguistic research has corroborated these findings, extending the validity of language biomarkers beyond English-speaking populations [21]. Studies conducted in diverse linguistic and cultural contexts, including Spanish-, Italian-, Dutch-, and Portuguese-speaking communities, have consistently identified similar language impairments [22]. These findings highlight the universality of language deficits in schizophrenia, suggesting that, despite linguistic and cultural variations, the core features of language impairment remain consistent.

Developments in machine learning and natural language processing have made it easier to create predictive models that can accurately distinguish between healthy individuals and patients with schizophrenia [23,24]. The definition of natural language processing is imprecise, as it broadly encompasses various computational techniques for analyzing and synthesizing human language but lacks a universally agreed-upon scope or detailed boundaries for its applications and methodologies [25]. As such, in the context of artificial intelligence, natural language processing is the interdisciplinary study that focuses on the communication between computers and human languages [25]. It entails the creation of models and algorithms that let computers comprehend, interpret, produce, and react to human language in a meaningful and practical way [25,26]. Typical examples of natural language processing cover a wide range of activities, such as text production, sentiment analysis, speech recognition, and language translation [27]. These models, for example, can attempt to predict the evolution from acute psychosis to chronic schizophrenia [28]. Such use of natural language processing could lead to early diagnosis and tailored treatment plans, but recent literature in this field agrees that more study is required to improve the models’ clinical applicability and interpretability. Considering the vast array of applications and uses of natural language processing and the potential uses of language as a biomarker in schizophrenia, there is a need to have a comprehensive literature review on the subject.

The objective of this study is to explore the applications of natural language processing in patients diagnosed with schizophrenia. A secondary aim is to assess the efficacy and limitations of these applications. It is hypothesized that the existing literature on this topic is relatively poor. Given the increasing research focus on predictive modeling using artificial intelligence algorithms, it is anticipated that the predominant application of NLP will be in predicting the progression of the disease. Additionally, due to the novel nature of these methodologies, it is hypothesized that their effectiveness remains under-researched and limited in terms of clinical interpretation and application [29].

## 2. Materials and Methods

### 2.1. Search Strategies

This review adhered to the standardized Preferred Reporting Items for Systematic Reviews and Meta-Analyses (PRISMA) guidelines for scoping reviews. A comprehensive scoping search was undertaken to identify recent studies across multiple electronic databases, including Medline, PubMed, Embase, and PsycInfo, covering the period from 2008 to 2023, as they are the main databases related to psychiatric research. The last 15 years was selected as the timeframe to encompass solely recent developments in the field of natural language processing. The search strategy utilized a combination of text words and subject headings, focusing on schizophrenia (e.g., schizophrenia, schizophrenic) and natural language processing (e.g., natural language processing, semantic analysis), ensuring alignment with the study’s objectives. Detailed search strategies can be found in Supplementary Material S1. The search methodology was expertly developed by an experienced librarian specialized in psychiatry (MD), and the searches were cross-validated using the Peer Review of Electronic Search Strategies (PRESS). No restrictions were applied regarding setting or geography, ensuring a comprehensive and unbiased search.

### 2.2. Study Eligibility

Studies were selected based on the following inclusion criteria: (1) the population of interest comprised patients diagnosed or research focused on schizophrenia or schizoaffective disorders; (2) the study utilized a natural language processing approach; (3) the natural language processing was applied to target a specific outcome. Studies were included regardless of whether they employed a single algorithm or multiple algorithms. Excluded from consideration were unpublished literature and studies employing artificial intelligence algorithms beyond the scope of natural language processing. The search was restricted to sources available in English and French.

### 2.3. Data Extraction

Data extraction was conducted using a standardized form in Microsoft Excel and was independently verified for consistency and integrity by two authors (A.D., A.H.). Any disagreements regarding the inclusion or exclusion of studies were resolved through mutual consensus. The systematically extracted information included authors, population (sample), primary uses (or intent) of the natural language processing algorithms, main outcomes, and limitations.

### 2.4. Quality Assessment

The quality assessment of the identified studies was carried out using the Newcastle–Ottawa Scale (NOS) for nonrandomized controlled studies and the Cochrane Risk of Bias Tool for randomized controlled trials [30,31]. The NOS is designed to evaluate cohort and case-control studies across the following three key areas: the selection of study groups, the comparability of these groups, and the ascertainment of either exposure or outcome [30]. Each area comprises specific criteria, and studies receive star ratings based on how well they meet these criteria, with a maximum score of nine stars indicating the highest quality [30].

For randomized controlled trials, the Cochrane Risk of Bias Tool provides a thorough framework to determine potential biases. This tool assesses the following seven domains: random sequence generation, allocation concealment, blinding of participants and personnel, blinding of outcome assessment, handling of incomplete outcome data, selective reporting, and other potential sources of bias [31]. Each domain is rated as a low, high, or unclear risk of bias according to set criteria.

In this literature review, studies were categorized by the authors based on their quality ratings. Those scoring 1–4 stars on the Newcastle–Ottawa Scale were identified as having a high risk of bias by the Cochrane tool were considered low quality. Studies with 4–6 stars were rated as moderate quality, while those achieving 7–9 stars or a low risk of bias were deemed of high quality.

## 3. Results

### 3.1. Description of Identified Studies

**Figure 1:**
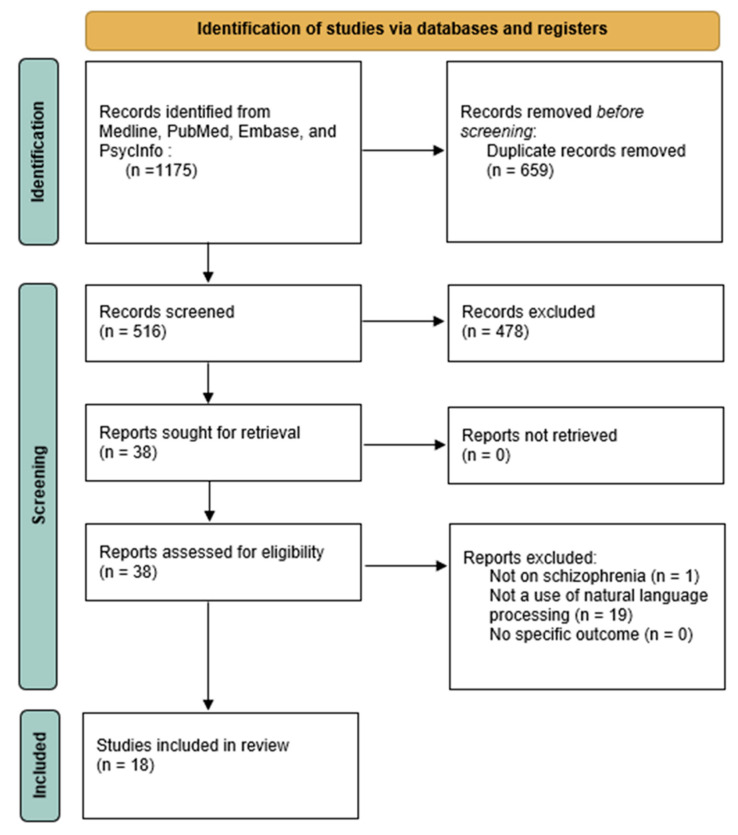
PRISMA flow diagram of identified studies.

The scoping review assessed studies at the intersection of schizophrenia and natural language processing. Initially, 516 eligible articles were identified after removing duplicates (n = 659). A total of 478 studies were excluded based on the first analysis of titles and abstracts, as they did not meet inclusion criteria. After a second round of abstract screening, 38 full-text articles were thoroughly evaluated, resulting in 20 exclusions. This left 18 studies for detailed analysis. A flowchart illustrating the inclusion process is provided in Figure 1, and specific details of the included studies are available in Table 1. The studies that met the inclusion criteria utilized natural language processing in various ways, with the most common applications being diagnostic and predictive modeling (n = 6), followed by specific linguistic phenomena (n = 3), speech and communication analysis (n = 3), social media and online content analysis (n = 3), clinical and cognitive assessment (n = 2), and linguistic feature analysis (n = 1).

### 3.2. Diagnostic and Predictive Modeling

The search approach identified a total of six studies that discussed the use of natural language processing for predictive and diagnostic modeling. In their study, Figueroa-Barra and colleagues analyzed 30 language variables from recorded interviews to perform an automated language analysis on 133 Spanish-speaking respondents, including healthy controls, patients with first-episode psychosis, and patients with persistent schizophrenia [34]. Their longitudinal analysis, which was mainly based on semantic coherence information, predicted the diagnosis of schizophrenia in first-episode psychosis patients with a 77.5% accuracy [34]. Their cross-sectional analysis demonstrated that language features could distinguish with an 85.9% accuracy between healthy controls and patients diagnosed with schizophrenia [34].

In another study, Voppel and colleagues used acoustic and semantic data taken from semi-structured interviews to assess speech from 94 people with schizophrenia spectrum disorders and 73 healthy controls [35]. In comparison to individual classifiers for acoustic (81% accuracy) and semantic (80% accuracy) features, the combined machine learning classifier—which combined acoustic and semantic features—achieved an 85% accuracy in differentiating schizophrenia spectrum disorder from healthy controls [35]. This suggests that combining these domains captures complementary aspects of speech affected by schizophrenia spectrum disorder.

Additionally, a study assessed the feasibility of gathering speech samples from 47 individuals with severe mental illness over a period of 4–14 months using a mobile intervention called MyCoachConnect, and then evaluating lexical and acoustic aspects to follow clinical states [36]. Their findings demonstrated that, when compared to provider global assessment ratings, individually trained models had a strong correlation (rho = 0.78, *p* < 0.001), suggesting the potential value of speech features as objective markers for tracking mental health conditions like schizophrenia in a community-based clinical setting [36]. Similarly, Bedi and her colleagues used baseline interviews to extract syntactic and semantic data from 34 young people who were clinically at high risk of psychosis through automated speech analysis [37]. With a 100% accuracy, the analysis predicted the onset of psychosis later, outperforming conventional clinical assessments by a significant margin [37]. Additionally, it showed a correlation between prodromal symptoms and speech features, indicating the potential value of automated speech analysis as a tool for early psychosis detection [37]. Another study collected 22 speech samples and correlated linguistic aspects with clinical assessments of psychotic symptoms in order to investigate the use of natural language processing to detect disorganized speech in seven hospitalized schizophrenia patients [38]. The findings showed that, whereas lower content density and more repetitions predicted good symptoms, reduced lexical richness and syntactic complexity were suggestive of negative symptoms [38]. The authors concluded that natural language processing has the capacity to quantify psychotic symptoms objectively in acute clinical settings, as demonstrated by their machine learning models that can accurately predict symptom severity up to 82% of the time [38].

Lastly, a recent study by Rezaii and colleagues used machine learning to assess the latent content and semantic density of speech from 40 individuals who were clinically high risk for psychosis [39]. They did this by comparing the speech content with a sizable corpus and measuring semantic richness using vector unpacking. It is possible for these speech characteristics to be reliable predictors of future psychosis, as evidenced by the 93% accuracy with which the combination of poor semantic density and higher discussion about voices and sounds predicted conversion to psychosis in the training set and 90% in the holdout set [39].

The quality of the identified studies for the use of natural language processing ranged from moderate to high, with the main limitation being the small sample sizes used to train the models.

### 3.3. Specific Linguistic Phenomena

Three studies emphasized how particular linguistic occurrences might be extracted through natural language processing to gain a better understanding of the characteristics of schizophrenia or diseases resembling schizophrenia. In one of the studies that was found, machine learning algorithms were used to compare the syntactic and semantic analysis of speech during interviews in 70 adolescents, 35 of whom had prodromal psychosis and the remaining 35 were healthy controls [44]. There were notable variations between the two groups, with the prodromal group using fewer nouns, pronouns, conjunctions, adjectives, prepositions, and proper nouns, as well as less coherence overall [44]. The authors support that these findings could serve as indicators for the early detection of psychosis. In another study, Parola and colleagues used a decision tree model to uncover characteristics that differentiate the two groups of 67 participants, 32 with schizophrenia and 35 healthy controls, by applying a multimodal assessment of communicative-pragmatic abilities [45]. Linguistic irony and breaking Gricean maxims emerged as the most significant aspects of the 82%-accurate model, indicating that these pragmatic deficiencies are important markers of schizophrenia [45]. Lastly, a different study including a bigger sample size examined how well 228 patients with first-episode psychosis, including those with affective and non-affective psychosis, and 70 healthy controls understood nonliteral language [46]. The study’s authors found that, particularly in open-ended tasks, patients with affective and non-affective psychosis performed worse than healthy controls when it came to comprehending metaphors and idioms [46]. They also suggested that deficiencies in nonliteral language comprehension could be an early indicator of psychosis.

The quality of the phenomena identified for this use also ranged from moderate to high. The main limitations included the small sample sizes and the heterogeneity of clinical profiles of the patients diagnosed with schizophrenia.

### 3.4. Speech and Communication Analysis

Another primary usage that the literature search revealed was the analysis of speech content. Such use was reported in three investigations. The first study that was found assessed the test–retest reliability and generalizability of automated speech organization and content measures in 47 people over a one-year period and 101 participants with schizophrenia over a six-month period [47]. The results demonstrated that speech organization measures had fair-to-good reliability, while speech content measures had fair reliability. Moreover, there were notable differences in speech indices according to education, income, and race, underscoring the necessity of taking demographic factors into account when using these automated measures [47]. The second study used video “selfies” to evaluate the validity and reliability of natural language processing metrics to diagnose paranoia in 35 patients with bipolar disorder or schizophrenia over the course of a week [48]. The integrated measure of paranoia showed strong convergent validity and test–retest reliability, but it also indicated demographic biases, with female and white subjects showing higher criterion validity [48]. Finally, a study by Gargano and colleagues employed a systematic procedure to assess the micro- (lexicon, morphology, syntax) and macro-linguistic (discourse coherence, pragmatics) levels of the narrative discourse in 133 patients with first-episode psychosis (95 non-affective, 38 affective) and 133 healthy controls [49]. With a machine learning model achieving a 76.36% accuracy in classifying first-episode psychosis patients versus healthy controls based on micro- and macro-linguistic levels, the results highlighted the utility of language features as potential diagnostic markers [49]. The patients’ significant language production deficits were found in both micro- and macro-linguistic domains. The studies identified for this use were of high quality, also reporting small sample sizes and that natural language processing approaches are time-consuming.

### 3.5. Social Media and Online Content Analysis

Three of the identified studies concerned content analysis of social media posts. A systematic review that focused on 7 out of 93 studies that were found to meet the inclusion criteria examined the use of social media data in conjunction with artificial intelligence natural language processing algorithms to diagnose and track psychotic illnesses [41]. Their research revealed that, although social media data show promise for diagnosing and tracking schizophrenia, the studies’ quality varied and they encountered issues like small sample sizes and restricted access to clinical diagnostic data, which made more research with better methodologies necessary to fully realize these tools’ potential [41].

The second study found in the literature review collected 60,009 posts from the Reddit subreddit dedicated to schizophrenia and 425,341 posts from six other subreddits related to non-mental health issues [42]. The study employed machine learning to identify schizophrenia from the social media material. The potential of social media analysis for the detection of schizophrenia is highlighted by the random forest algorithm’s 96% accuracy in identifying posts linked to the disorder [42]. Significant linguistic markers, such as an increase in the use of third-person plural pronouns and words that convey negative emotions, were also identified. Similarly, the last study for this use analyzed Twitter (now X) data from 671 users who self-disclosed their illness to uncover social media signs of schizophrenia using a human–machine partnered strategy [43]. A classifier that identified users with schizophrenia with an 88% accuracy using linguistic features was created by combining clinician assessments with machine learning to separate genuine disclosures from noisy data [43]. This highlights the significance of combining clinical expertise with computational methods to improve the accuracy of mental health diagnoses on social media.

The quality of the studies ranged from moderate to high, with the main limitation being that assumptions are made regarding potential users having schizophrenia when assessing the online content of social media, as there is no way to counter-verify self-claims of being diagnosed with schizophrenia.

### 3.6. Clinical and Cognitive Assessment

Two of the identified studies were relevant to clinical and cognitive assessment. Using CoVec, an automated language tool, Ku and colleagues tested semantic coherence in a semantic fluency task completed by 197 first-episode psychosis patients and compared those with and without derailment and tangentiality [32]. Coherence-5 and Coherence-10 levels were significantly lower in derailed patients, according to the results, indicating the possibility of automated methods like CoVec to objectively detect formal thought problems in schizophrenia [32]. Another type of clinical assessment, concentrating on the Theory of the Mind, utilized machine learning, specifically Bayesian network analysis, to characterize the relationships between cognitive functions, Theory of Mind, and pragmatic abilities in 32 individuals with schizophrenia and 35 healthy controls [33]. The Bayesian network classifier achieved a 95.5% accuracy in distinguishing patients from controls, identifying linguistic pragmatic ability as the most significant factor in classification, suggesting that pragmatic impairment is a core dysfunction in schizophrenia [33]. Quality assessment of both studies was deemed high. Small sample sizes were reported as limitations in both studies when assessing the models.

### 3.7. Linguistic Feature Analysis

To better understand the language associated with self-experience, a study by Chan and colleagues examined the use of natural language processing to analyze autobiographical narratives from 167 patients with schizophrenia or schizoaffective disorder and 90 healthy controls [40]. A machine learning classifier trained on these features achieved an area under the curve of 0.80, indicating its potential for differentiating between schizophrenia and healthy controls and highlighting significant relationships between self-experience language features and clinical symptoms [40]. The analysis showed that topics related to self-experience were significantly more expressed in patients with schizophrenia. The quality of the study was evaluated as high and the authors reported major limitations of their approach such as a validated measure of self-disturbance not being used; comprehensive speech signals (including speech sounds and facial expressions) not being sufficiently evaluated; disruptions in self-experience being more common in trauma, with a disproportionate number of veterans in the schizophrenia group; text embedding only revealing the frequency of phrases, not their relationships; no information on the rates of trauma or comorbid disorders.

## 4. Discussion

The main objective of this scoping review was to identify the main uses of natural language processing in the field of schizophrenia. Six major uses were identified by the literature review, and using natural language processing for diagnostic and predictive modeling was the most prevalent. The authors of the identified studies reported many limitations, most of them highlighting the small sample sizes used to train the models, which may hinder their performances.

With the rise of machine learning in the field of healthcare to help clinicians in predicting outcomes of mental health disorders, as well as predicting the course of mental illnesses, it is unsurprising that the most prevalent use of natural language processing in the field of schizophrenia is about diagnostic and predictive modeling [50].

Considering the impairments of language observed in patients suffering from schizophrenia, many of the uses identified focused on linguistic, speech analysis, and social media contents, as they encompass various heterogeneous components of language. A recent study by de Boer and colleagues investigated the relationship between language characteristics, schizophrenia diagnosis, symptom severity, and white matter integrity using spontaneous speech recordings and diffusion tensor imaging in 26 schizophrenia patients and 22 healthy controls [8]. The analysis classified schizophrenia with 89% sensitivity and 82% specificity, found language disturbances linked to negative symptom severity, and showed that computational language measures predicted white matter integrity, indicating the potential clinical and biological validity of quantitative language analysis in schizophrenia research [8]. The performance of their classification is like the natural language processing models identified in this literature review. Furthermore, another recent study examined language dysfunction in schizophrenia by assessing neural tracking using electroencephalography in response to naturalistic speech stimuli [7]. Their results indicated that schizophrenia patients exhibited impaired neural tracking of speech, particularly in theta-band oscillations, suggesting deficits in both auditory–sensory and abstract linguistic processing, which may contribute to the language-related symptoms observed in schizophrenia, and this should be further studied [7]. Using these findings and natural language processing modeling could help clinicians in their assessment and monitoring of patients diagnosed with schizophrenia. The study identified also aligns with recent literature reporting that patients with schizophrenia (or schizoaffective disorder) had intact lexical processing but exhibited significant deficits in sentence comprehension, particularly in syntactic processing, which correlated with the severity of formal thought disorder symptoms, highlighting a potential link between language comprehension and thought disorder in schizophrenia [51]. As an example, the authors reported that, in schizophrenia, word form recognition remained intact, suggesting normal lexical processing [51]. With increasing severity of formal thought disorder, there was a propensity to impute word meanings based on phonetic similarities, although single-word synonym recognition was not significantly affected [51].

While there is a vast array of literature on the cognitive impairments observed in patients suffering with schizophrenia, this literature review highlighted a small number of studies that used natural language processing for assessing such impairments. As an example, in the field of dementia, notably Alzheimer’s Disease, natural language processing is being studied extensively, as it may be a good avenue to screen cognitive decline. For instance, a recent study investigated the Pre-screening Tool for Language and Cognition Assessment Model (PST-LCAM), using a combination of psycholinguistic and cognitive adequacy (PCA) of Role and Reference Grammar (RRG) mapped to speech production parameters from the DementiaBank dataset [52]. The main results show that the PST-LCAM effectively correlates with clinical assessments, demonstrating consistent indicators of cognitive decline as validated against the Global Deterioration Scale (GDS), suggesting its potential utility for the early detection of dementia [52]. Conducting further research on the applications of natural language processing to assess cognitive impairments in patients with schizophrenia could yield potential therapeutic or preventive approaches.

### Limitations

The heterogeneity of diagnostic criteria for schizophrenia is a key concern that is not addressed in most of the examined research, as Table 1 shows. Concerns regarding the results’ generalizability are further raised by the absence of external validation in populations that differ from the training sample, such as those belonging to various nations. Performance comparisons were not carried out because of the variability of the listed research and the different measures utilized to evaluate precision and validate the natural processing algorithms. The studies identified also reported various ways of diagnosing schizophrenia or related psychotic disorders which may affect the external validity of these results.

## 5. Conclusions

Considering the importance of language and its impairment in patients diagnosed with schizophrenia, this literature review offered a first overview of natural language processing applications in this field. The review identified six primary natural language processing uses in schizophrenia. The main uses encompassed diagnostic and predictive modeling, which is the use of natural language processing to predict schizophrenia or related psychotic disorders. It also reported specific linguistic phenomena as attempting to model how linguistic characteristics can help in better understanding schizophrenia. A more specific speech and communication analysis made by a natural language processing model reported how speech organization can be used to understand schizophrenia as compared to healthy controls. Social media and online content analysis combined with natural language processing enabled the early identification of schizophrenia using verbatims on social media platforms. The last main two uses were the use of clinical and cognitive assessment as well as linguistic feature analysis to further comprehend the clinical presentations of schizophrenia. However, the field’s novelty is underscored by the small sample sizes and lack of clinical validation in many studies. Future research should focus on larger, diverse samples and practical clinical applications to confirm the utility of natural language processing in schizophrenia diagnosis and treatment.

## Figures and Tables

**Table 1:** Systematic review study selection detailed results.

Authors	Population (Type of Participants, N)	Main Use of NLP	Outcomes	Limitations	Quality
Ku, B. S., et al. (2021) [32]	N = 197. Patients presenting with FEP.	Clinical and Cognitive Assessment	Patients with derailment produce significantly fewer words than those without derailment. First-episode psychosis patients with moderate-to-severe derailment have a lower Coherence-5 score (0.554) compared to those without derailment (0.570), with a small-to-medium effect size (d = 0.27).	The study has several limitations: SAPS is not a fully accurate or gold-standard method for evaluating FTD due to the subjectivity of the clinician administering the scale, and the three different CoVec output measures are inter-related, measuring the same phenomenon with slightly different approaches.	High
Parola, A., et al. (2020) [33]	N = 67. A total of 32 individuals with SCZ and 35 HCs (Italy).	Clinical and Cognitive Assessment	The model’s sensitivity showed that all patients with SCZ were classified correctly, with high overall accuracy and good precision, resulting in very few false positives. Pragmatic linguistic ability was identified as the most important factor in distinguishing between SCZ patients and HCs. SCZ was associated with poor performance on tasks involving Theory of Mind, selective attention, extra-linguistic abilities, planning, and inhibition. There was a less clear association for paralinguistic abilities and cognitive flexibility, where patients showed a wider range of performance values.	Relatively small sample size.	High
Figueroa-Barra, A., et al. (2022) [34]	N = 133 (HC = 49; FEP = 40; Chronic SCZ = 44). All exclusively Spanish-speaking subjects from Chile.	Diagnostic and Predictive Modeling	Using the top ten ranked variables, the model’s accuracy in differentiating between groups was 80.97% (HC vs. SCZ), 85.93% (HC vs. FEP + SCZ), and 91.11% (HC vs FEP) with a random forest classifier. To evaluate FEP conversion to SCZ, accuracy was measured. Results were poor with only demographic information (43.33%) but improved with PANSS information (65.83%). PANSS allowed for a 67.5% prediction accuracy. Language-only provided a 75.83% accuracy. Combining all information and using the top ten features resulted in a 77.5% accuracy for predicting if an FEP patient would have a confirmed SCZ diagnosis.	Use of exclusively Chilean HCs, self-reported comorbidities like drug abuse, and differing demographic variables between healthy and psychotic subjects, which may introduce potential bias. There was no record of refusals at recruitment. The random forest model used for analysis has a simple and broad interpretation, and the study’s limited sample size may lead to overfitting. Additionally, the longitudinal analysis classes were unbalanced.	High
Voppel, A. E., et al. (2023) [35]	N = 167. A total of 94 patients with SCZ spectrum disorder (SSD) and 73 HCs.	Diagnostic and Predictive Modeling	Acoustic classifier: 81% accuracy, 89% sensitivity, 70% specificity, AUC-ROC 0.82. Semantic classifier: 80% accuracy, 81% sensitivity, 78% specificity, AUC-ROC 0.83. Combined classifier: 85% accuracy, 92% sensitivity, 79% specificity, AUC-ROC 0.88.	Significant differences in years of education between groups, possible audio contamination from background noise, low test–retest validity for acoustic features, and the use of cross-validation to estimate the generalizability of the models.	High
Arevian, A. C., et al. (2020) [36]	N= 47. Participants recruited from a community-based mental health clinic for adult patients with serious mental illness.	Diagnostic and Predictive Modeling	Using an individually trained algorithm, prediction models showed a high correlation (up to 0.78) between predicted and actual clinical states based on providers’ global assessment ratings. There was little correlation between individuals regarding which speech features correlated with their clinical state, suggesting that word choice patterns related to mental illness/wellness may be specific to individuals. Both population-based and individualized approaches can inform computational methods using behavioral markers. Statistically significant correlations were found between the model and actual scores for the summary, depression, and self-harm sub-scores of the BASIS-24, and the mental health sub-score of the SF-12. No significant correlations were found for four of the six BASIS-24 sub-scores or the physical health sub-score of the SF-12.	Variability and subjectivity in global assessment ratings due to clinician differences, a small sample size that does not account for participant characteristics, an inability to determine the strengths or weaknesses of features and algorithms for symptom-specific states or differences compared to healthy volunteers, and a predictive model that does not significantly predict the physical health subscale.	Moderate
Bedi, G., et al. (2015) [37]	N = 34. Participants were help-seeking youths aged 14–27; referred by school, clinicians or self-referred through the Center of Prevention and Evaluation website.	Diagnostic and Predictive Modeling	Baseline speech recordings and transcriptions accurately predicted the transition to psychosis in a high-risk clinical group. Automated analysis outperformed clinical ratings, showing that automated speech analysis can enhance predictive accuracy beyond expert clinical opinion.	Small sample size.	High
Jeong, L., et al. (2023) [38]	N = 7. Patients admitted to a psychiatric inpatient unit between 2019 and 2021, with diagnostic of SCZ and psychosis as the main reason of admission.	Diagnostic and Predictive Modeling	Participants with severe symptoms of poverty of speech, content, and social inattentiveness showed reduced lexical richness and syntactic complexity, with lower Honore’s statistics, shorter word lengths, more high-frequency words, shorter sentences and clauses, and fewer coordinations and prepositions. Those with higher derailment and pressure of speech used more words, clauses, and longer sentences but had lower type–token ratios and content density. Lower BERT next-sentence probability scores were linked to severe derailment, illogicality, and circumstantiality. Machine learning models predicted alogia, illogicality, poverty of speech, social inattentiveness, and global TLC scores with up to an 82% accuracy (0.82 F1 score). Preliminary results show that NLP can predict symptom severity from speech records.	The study has several limitations: a small sample size, reliance on the attending psychiatrist’s subjective judgment as the gold standard, and the use of third-party manual transcription services to convert speech recordings into text.	High
Rezaii, N., et al. (2019) [39]	N = 40. Participants of the North American Prodrome Longitudinal Study (NAPLS) at Emory University. A total of 30 from the second phase (NAPLS-2) and 10 from the third phase (NAPLS-3).	Diagnostic and Predictive Modeling	Semantic density predicted conversion to psychosis in 80% of cases (60% sensitivity, 100% specificity). Latent semantic content (VOICES) predicted conversion in 70% of cases (40% sensitivity, 100% specificity). Combining semantic density and VOICES predicted conversion in 93% of cases (86% sensitivity, 96% specificity).	The small number of participants, insufficient variety in neuropsychiatric disorders studied, and no inclusion of HCs weakens the generalizability and reliability of the results.	High
Chan, C. C., et al. (2023) [40]	N = 257; 167 patients with SCZ or schizoaffective disorder, 90 HCs.	Linguistic Feature Analysis	Several markers related to self-experience emerged as top features differentiating SCZ from HCs. “Self-experience and Agency” was higher in SCZ. SCZ patients used a more negative emotional tone, spoke less about “Burden”, and used more negative emotional words. Language related to self-experience strongly correlates with clinical symptoms.	Text embedding only reveals the frequency of phrases, not their relationships; there were not enough unmedicated patients to evaluate the effect of medication properly; no validated measure of self-disturbance was used; comprehensive speech signals (including speech sounds and facial expressions) were not sufficiently evaluated; disturbances in self-experience are also prevalent in trauma, with a disproportionate number of veterans in the SCZ group; there was no data on the rates of trauma or comorbid disorders.	
Lejeune, A., et al. (2022) [41]	A total of 7 studies included, published in the United States (5) and Korea (2) between 2015 and 2021. The samples size varied between 51 and 265,396 participants.	Social Media and Online Content Analysis	Social media data may be utilized for a variety of purposes in the treatment of individuals with schizophrenia, such as post-first psychotic episode patient monitoring.	A small number of included studies, most of which did not use clinical diagnostic data, and limitations in both the methodology and the choice of machine learning algorithms.	Moderate
Bae, Y. J., et al. (2021) [42]	Large corpus of social media posts collected from the Reddit website between September 2016 and September 2020. N = 13,156 (posts from Reddit sub-communities for SCZ); N = 247,569 (posts from non-mental-health-related subreddits).	Social Media and Online Content Analysis	Classification: A random forest machine learning model achieved a a high accuracy of 96% (94% recall, 98% precision, 96% F1-score, and 0.97 AUC) in distinguishing between SCZ and control groups. Linguistic characteristics of SCZ: Posts from individuals with SCZ showed significant linguistic differences compared to control participants. SCZ posts had a lower word count (WC), fewer first-person singular (FPS) and third-person singular (TPS) pronouns, past tense, and positive emotion words. They had higher second-person (SP) pronouns, third-person plural (TPP) pronouns, impersonal pronouns, present tense, and negative emotion words. Topic detection and comparison: The SCZ subreddit focused more on diagnosis (diagnostic), symptoms (Sx), treatment (Tx), and the nature of the disorder. Control subreddits discussed family, friends, social relationships, and general topics more frequently. Increased use of symptom-related words and decreased occurrence of positive general topics characterized the language in the SCZ subreddit.	It focuses on identifying specific textual information in SCZ rather than examining various types of mental disorders, lacks evidence that SCZ subreddit users have an actual diagnosis, and users of the SCZ subreddit may not be representative of the broader population, introducing selection bias. Only commonly used machine learning classifiers were evaluated, and the findings may be limited to Reddit users and might not generalize to users of other platforms.	Moderate
Birnbaum, M. L., et al. (2017) [43]	N = 292. A total of 146 Twitter users in the SCZ group and 146 Twitter users in the control group.	Social Media and Online Content Analysis	SCZ group: More frequent use of first-person pronouns and words related to biological processes (body, health), less frequent use of words related to “friends.” Subtle language changes may also be linked to SCZ. The classifier agreed with experts in removing inauthentic samples but was overly inclusive in labeling true SCZ cases.	The authors report various limitations: it is impossible to confirm a diagnosis of SCZ via Twitter alone, symptoms of psychosis are not limited to SCZ, access was restricted to public profiles, the classifier was developed using only linguistic variables, and the findings may be limited to Twitter users and may differ from users of other platforms.	High
Malik, K., et al. (2023) [44]	N = 70. A total of 35 participants in the prodromal group (based on the Prodromal Questionnaire-Brief, Indonesian version); 35 participants in the healthy control group. Participants were aged 14–19 years.	Specific Linguistic Phenomena	Several features significantly distinguish prodromal psychosis adolescents from adolescents without prodromal psychosis, including decreased semantic coherence and word complexity in sentences. Syntactic analysis showed no significant difference in speech production between the two groups. There were differences in word-use frequency in the prodromal psychosis group compared to the group without prodromal psychosis. While not many syntactic and semantic features were statistically significant, linguistic trends similar to those in SCZ patients were found, suggesting the need for a separate model to improve prediction accuracy. The highest accuracy achieved by various classifiers was 57%, with a standard deviation of 3.2% using a random forest classifier.	The authors reported everal limitations: a small sample size, potential selection bias from only including participants who responded, and the severity of prodromal psychosis symptoms possibly preventing some from responding. Substance use was eliminated as a confounding factor for adolescent psychosis risk. Prodromal subjects showed subtle speech disorganization, indicating a need for improved predictive models like the tangentiality prediction model. There were imbalances in variables, such as age, psychiatric history, and family history between prodromal and healthy control participants, as well as in the proportion of coherent and incoherent phrase segments analyzed.	High
Parola, A., et al. (2021) [45]	N = 67. A total of 32 individuals with SCZ and 35 HCs (Italy).	Specific Linguistic Phenomena	The model achieved an accuracy of 0.821 (SD = 0.118), a sensitivity of 0.758 (SD = 0.285), a precision of 0.910 (SD = 0.151), a specificity of 0.900 (SD = 0.175), and an AUC of 0.894 (SD = 0.143). Linguistic irony was the most important factor for classifying individuals as either SCZ patients or HCs.	Sample is relatively small. Heterogeneity of clinical profiles of patients with SCZ disorder not considered. Pragmatic ability can be measured in different ways; communicative features found to be the most informative to classify participants would need to be confirmed in further studies across different pragmatic tasks and contexts.	Moderate
Perlini, C., et al. (2018) [46]	N = 228. FEP AP = 60; FEP NAP = 168. FEP outpatients recruited from 117 public community mental health centers in Northern Italy. Control group = 70, with no DSM-IV axis I disorder.	Specific Linguistic Phenomena	After adjusting for cognitive measures (flexibility, working memory, IQ), only OPEN task results remained statistically significant, showing similar results for both patient groups compared to HCs.	No assessment of ToM in participants (partly related to comprehension of figurative speech). No observation of possible variations in participants’ speech over time, given the cross-sectional nature of the study. Effects of pharmacotherapt not taken into account. DSM-IV axis II disorders in the control group not assessed or excluded (e.g., schizotypal traits). Lack of a comprehensive evaluation of different pragmatic linguistic aspects (prosody, figures of speech, etc.).	high
Minor, K. S., et al. (2023) [47]	N = 101 (for baseline to six months sample). N = 47 (for baseline to 1 year sample). N total = 148. All participants were enrolled in ≥one of three studies at a Midwestern Veterans Affairs Medical Center (VA), with parent studies having taken place from 2004 to 2016.	Speech and Communication Analysis	Speech content indices generally met fair reliability standards, while speech organization indices were mostly fair to good. Changes in speech indices from baseline to six months rarely varied based on demographics. Speech indices showed the most differences in race, income, and education, but more convergence for age and gender. Regarding replicability, both speech content and organization indices generally did not meet the good test–retest reliability standards of other instruments.	Use of IPII may produce speech that is difficult to replicate, a small number of women participants complicates the analysis of gender effects, and a large sample size of older individuals makes it hard to differentiate between the effects of long-term antipsychotic use and the prolonged effects of the illness.	High
Cohen, A. S., et al. (2022) [48]	N = 35. A total of 31 patients with DSM-5 diagnostic of SCZ, and 4 patients with DSM-5 diagnostic of bipolar disorder.	Speech and Communication Analysis	The integrated NLP-based measure of paranoia in this study was reliable over a week, showed good alignment with our criterion and related measures, and diverged from global measures of negative affect and psychopathology. It had higher criterion validity for white and female participants than for black and male participants. This highlights the need for thorough psychometric evaluations for NLP measures of psychosis, considering demographic biases to avoid missing critical issues.	Sample size is modest and demographically constrained, video data are incomplete for many participants, the sample consists exclusively of participants with serious mental issues, suggesting the need for a community sample with a wider spectrum of symptoms for future studies, and the epoch is limited to a week.	High
Gargano, G., et al. (2022) [49]	N = 266. A total of 133 FEP (95 non-affective FEP; 35 affective FEP) and 133 HCs.	Speech and Communication Analysis	FEP patients show significant deficits in micro-level speech production, including intraphrasal discourse construction. They use fewer lexical fillers, have a lower speech rate, and shorter utterances compared to HCs. Their narratives have a lower percentage of syntactic completeness. Both affective and non-affective FEP patients exhibit significant impairments in speech rate and mean length of utterances compared to HCs, indicating that all psychotic patients have some impairment in productive aspects of phrasal construction. No significant difference was found between FEP-NA and FEP-A in language production. FEP patients performed worse than controls on neuropsychological tasks (verbal IQ, n-back, SOA). The model using only language production variables had a prediction accuracy of 76.36%. Machine learning results showed that GAF alone predicted FEP and HC groups with a 97.90% accuracy, while neuropsychological measures had a predictive power of 99%	The study has several limitations: all of the participants were outpatients with only moderate impairment (GAF scores), no significant differences in language production impairments between FEP-A and FEP-NA were found, differences in sample sizes between FEP-A and FEP-NA (38 vs. 95), and the linguistic analysis method used was time-consuming, limiting its clinical applicability.	High

Acronyms: AP: antipsychotic, AUC: area under the curve, BASIS: Behavior and Symptom Identification Scale, BERT: Bidirectional Encoder Representations from Transformers, DSM: Diagnostic and Statistical Manual of Mental Disorders, FEP: first-episode psychosis, FPS: first-person singular, FTD: formal thought disorder, GAF: Global Assessment of Functioning, HC: healthy control, II: Intelligence Index, IPII: Information Processing Index II, IQ: intelligence quotient, IV: independent variable, NA: not applicable, NAP: non-affective psychosis, NAPLS: North American Prodrome Longitudinal Study, NLP: natural language processing, OPEN: open-ended task, PANSS: Positive and Negative Syndrome Scale, ROC: receiver operating characteristic, SAPS: Scale for the Assessment of Positive Symptoms, SCZ: schizophrenia, SD: standard deviation, SF: short-form (related to health surveys, e.g., SF-12), SOA: stimulus-onset asynchrony, SP: second-person, SSD: schizophrenia spectrum disorder, TLC: Thought, Language, and Communication Scale, TPP: third-person plural, TPS: third-person singular, VA: Veterans Affairs, VOICES: Latent Semantic Content Model, WC: word count.

## Data Availability

Not applicable.

## Supplementary Materials

The following supporting information can be downloaded at: https://www.mdpi.com/article/10.3390/jpm14070744/s1, Supplementary material S1: Concept plan and search strategies.
