# Supplementary material for: Natural Language Processing and Schizophrenia: A Scoping Review of Uses and Challenges
Source: J Pers Med. 2024 Jul 12;14(7):744. doi: 10.3390/jpm14070744 (PMC11278236; doi:10.3390/jpm14070744)
Supplement: Supplementary file 1 [file jpm-14-00744-s001.zip › jpm-3094021-supplementary.pdf]

Supplementary Material

Deneault A, Dumais A, Désilets M, Hudon A. Natural language processing and Schizophrenia: A Scoping Review of Uses and Challenges

Supplementary material S1: Concept plan and search strategies

Concept plan

Natural language processing / Semantic analysis	Patients diagnosed with schizophrenia or psychosis disorders
<u>Subject Headings :</u><br>Natural Language Processing [Mesh]<br><br><u>Keywords :</u><br>natural language processing<br>semantic analysis<br>speech analysis processing<br><br>language<br>linguistic<br>AND<br>Automated<br>Automatic<br>Machine<br>Computational	<u>Subject Headings :</u><br>Schizophrenia Spectrum and Other Psychotic Disorders [Mesh]<br>Schizophrenic Language [Mesh]<br><br><u>Keywords:</u><br>Schizophrenia<br>Schizophrenic disorder(s)<br>Schizophrenic patient(s)<br>Schizoaffective<br>Schizoaffective Disorder(s)<br>Schizoaffective patient(s)<br>Schizo-affective<br>Schizo-affective disorders(s)<br>Schizo-affective patient(s)<br>Psychotic<br>Psychosis<br>Psychoses

<u>Concept 1 (Example in Pubmed)</u>
"Natural Language Processing"[MeSH Terms] OR "Natural Language Processing"[TIAB:~3] OR "semantic analysis"[TIAB:~3] OR "speech analysis processing"[TIAB:~3] OR ((language[TIAB] OR linguistic[TIAB]) AND (automated[TIAB] OR automatic[TIAB] OR machine[TIAB] OR computational[TIAB]))

<u>Concept 2 (Example in Pubmed)</u>
"Schizophrenic Language"[Mesh] OR ("Schizophrenia Spectrum and Other Psychotic Disorders"[Mesh]) OR ("schizo*"[TIAB]) OR (psychotic[TIAB]) OR (psychosis[TIAB]) OR (psychoses[TIAB])

<u>Search with keywords</u>

Concept 1
(Natural language or semantic or speech) ADJ3 (processing or analysis)
OR ((language OR linguistic) AND (automated OR automatic OR machine OR computational)

Concept 2
"schizo*" OR psychotic OR psychos?s

<u>PubMed</u>

(("Natural Language Processing"[MeSH Terms] OR "Natural Language Processing"[Title/Abstract:~3] OR "semantic analysis"[Title/Abstract:~3] OR "speech analysis processing"[Title/Abstract:~3] OR (("language"[Title/Abstract] OR "linguistic"[Title/Abstract]) AND ("automated"[Title/Abstract] OR "automatic"[Title/Abstract] OR "machine"[Title/Abstract] OR "computational"[Title/Abstract]))) **AND** ("Schizophrenic Language"[MeSH Terms] OR "Schizophrenia Spectrum and Other Psychotic Disorders"[MeSH Terms] OR "schizo*"[Title/Abstract] OR "psychotic"[Title/Abstract] OR "psychosis"[Title/Abstract] OR "psychoses"[Title/Abstract]) AND 2008/01/01:2023/12/31[Date - Publication]) AND (english[Filter] OR french[Filter])

Results: 251

<u>MEDLINE(R)</u>

1 Natural Language Processing/

2 ((Natural language or semantic or speech analysis) adj3 (processing or analysis)).ab. or ((Natural language or semantic or speech analysis) adj3 (processing or analysis)).ti.

3 ((language or linguistic) and (automated or automatic or machine or computational)).ab. or ((language or linguistic) and (automated or automatic or machine or computational)).ti.

4 1 or 2 or 3

5 exp "Schizophrenia Spectrum and Other Psychotic Disorders"/

6 Schizophrenic Language/

7 (schizo* or psychotic or psychos?s).ab. or (schizo* or psychotic or psychos?s).ti.

8 5 or 6 or 7

9 4 and 8

10 limit 9 to (yr="2008 -Current" and (english or french))

Results: 247

Embase

1 natural language processing/

2 ((Natural language or semantic or speech) adj3 (processing or analysis)).ti. or ((Natural language or semantic or speech) adj3 (processing or analysis)).ab.

3 ((language or linguistic) and (automated or automatic or machine or computational)).ab. or ((language or linguistic) and (automated or automatic or machine or computational)).ti.

4 1 or 2 or 3

5 exp schizophrenia spectrum disorder/

6 exp psychosis/

7 (schizo* or psychotic or psychos?s).ab. or (schizo* or psychotic or psychos?s).ti.

8 5 or 6 or 7

9 4 and 8

10 limit 9 to ((english or french) and yr="2008 -Current" and (article or article in press or "review"))

Results: 476

APA PsycInfo

1 natural language processing/ 1009

2 ((Natural language or semantic or speech analysis) adj3 (processing or analysis)).ab. or ((Natural language or semantic or speech analysis) adj3 (processing or analysis)).ti. 7811

3 ((language or linguistic) and (automated or automatic or machine or computational)).ab. or ((language or linguistic) and (automated or automatic or machine or computational)).ti. 6987

4 1 or 2 or 3 14079

5 exp psychosis/ 128046

6 (schizo* or psychotic or psychos?s).ab. or (schizo* or psychotic or psychos?s).ti. 183972

7 5 or 6 190368

8 4 and 7 390

9 limit 8 to (("0100 journal" or "0110 peer-reviewed journal" or "0120 non-peer-reviewed journal" or "0130 peer-reviewed status unknown") and journal article and (english or french) and yr="2008 -Current")

Results: 198
